# Exploring word memorability: How well do different word properties explain item free-recall probability?

**DOI:** 10.3758/s13423-020-01820-w

**Published:** 2020-10-15

**Authors:** Christopher R. Madan

**Affiliations:** School of Psychology, University Park, University of Nottingham, Nottingham, NG7 2RD UK

**Keywords:** Episodic memory, Animacy, Usefulness, Semantic properties, Verbal memory

## Abstract

What makes some words more memorable than others? Words can vary in many dimensions, and a variety of lexical, semantic, and affective properties have previously been associated with variability in recall performance. Free recall data were used from 147 participants across 20 experimental sessions from the Penn Electrophysiology of Encoding and Retrieval Study (PEERS) data set, across 1,638 words. Here, I consider how well 20 different word properties—across lexical, semantic, and affective dimensions—relate to free recall. Semantic dimensions, particularly animacy (better memory for living), usefulness (with respect to survival; better memory for useful), and size (better memory for larger) demonstrated the strongest relationships with recall probability. These key results were then examined and replicated in the free recall data from Lau, Goh, and Yap (*Quarterly Journal of Experimental Psychology, 71*, 2207–2222, 2018), which had 532 words and 116 participants. This comprehensive investigation of a variety of word memorability demonstrates that semantic and function-related psycholinguistic properties play an important role in verbal memory processes.

Some experiences are remembered better than others. While many studies have examined how different image properties can explain memorability of images (e.g., Bainbridge, Isola, & Oliva, 2013; Broers, Potter, and Nieuwenstein, 2018; Grühn & Scheibe, 2008; Isola, Xiao, Parikh, Torralba, & Oliva, 2014; Madan, Bayer, Gamer, Lonsdorf, & Sommer, 2018; Snodgrass & Vanderwart, 1980), our understanding of what makes a word more or less memorable is largely based on the relative influences of specific word properties—such as word imageability, frequency, and arousal—in studies where other properties are constrained. Though the use of word lists to study human memory has been a long-standing staple (Calkins, 1898; Kirkpatrick, 1894; Stoke, 1929), the literature on memorability for words is sparse (but see Christian, Bickley, Tarka, & Clayton, 1978; Rubin, 1980; Rubin & Friendly, 1986). Moreover, the generalizability of findings from image memorability are somewhat limited, as images tend to consist of many separable object ‘items’ (e.g., see Isola et al., 2014) and many images can map to a singular word (e.g., MOUNTAIN or SQUIRREL). Nonetheless, word stimuli have been common in the memory literature, as well as other areas of experimental psychology, for their ease in presenting to participants and ease for participants to report (e.g., relative to images or complex events). While exploring what makes a word memorable is of interest to memory researchers, it is also a question that bears relevance to those that study psycholinguistics, object knowledge, emotional processing, and others. Here, free-recall probability was calculated from a large-scale verbal memory study and compared with an array of lexical, semantic, and affective word properties to explore which properties best explain word memorability.

Many word properties—including word frequency, imageability, age of acquisition, arousal, and animacy—have been shown to relate to memory performance. In verbal memory studies, words are often selected such that words primarily vary along a specific dimension, such as word frequency or imageability, but other properties are matched between the word pools and then considered inconsequential. Some properties are related to their lexical features, such as the number of letters (better recall for short words; e.g., Baddeley, Thomson, & Buchanan, 1975; Frincke, 1968; Hulme, Suprenant, Bireta, Stuart, & Neath, 2004; Tehan & Tolan, 2007), number of syllables (better recall for fewer syllables; e.g., Baddeley et al., 1975; Hulme et al., 2004; Watkins, 1972), word frequency (better recall for high frequency; e.g., Gregg, 1976; Hall, 1954; Madan, Glaholt, & Caplan, 2010; Popov & Reder, 2019; Sumby, 1963), and orthographic neighbourhood size (better recall for more neighbours; e.g., Glanc & Greene, 2012; Jalbert, Neath, Bireta, & Surprenant, 2011; Jalbert, Neath, & Surprenant, 2011b). Other properties are related to their semantic features, such as age of acquisition (better recall for late acquired; e.g., Dewhurst, Hitch, & Barry, 1998; Morris, 1981), concreteness (better recall for high concreteness; e.g., Frincke, 1968; Madan et al., 2010; Paivio, Rogers, & Smythe, 1968; Stoke, 1929), animacy (better recall for living things [discussed in more detail in the Method section]; e.g., Bonin, Gelin, & Bugaiska, 2014; Bonin, Gelin, Laroche, Méot, & Bugaiska, 2015; Gelin, Bugaiska, Méot, & Bonin, 2017; Leding, 2019; Nairne, VanArsdall, Pandeirada, Cogdill, & LeBreton, 2013; Popp & Serra, 2016), number of features/semantic richness (better recall for higher number of features; e.g., Hargreaves, Pexman, Johnson, & Zdrazilova, 2012), and motoric properties (better recall for words referring to functional objects; Madan, 2014; Madan & Singhal, 2012; Montefinese, Ambrosini, Fairfeld, & Mammarella, 2013). Additionally, affective properties such as arousal and valence are also related to recall (better recall for high arousal and more extreme valence; e.g., Buchanan, Etzel, Adolphs, & Tranel, 2006; Kensinger & Corkin, 2003; Madan, Caplan, Lau, & Fujiwara, 2012; Madan, Scott, & Kensinger, 2019; Madan, Shafer, Chan, & Singhal, 2017). Moreover, several other word properties have only begun to be investigated in relation to memory, were also considered (e.g., with respect to human survival, danger, and usefulness). For instance, Leding (2019) recently demonstrated an independent and additive effect of a word’s associated threat, beyond memory effects related to animacy (e.g., ANTELOPE and ALLIGATOR, are both animate, but differ in threatening; DIPLOMA and DYNAMITE are both inanimate, and also differ in threat). While many word properties are correlated with each other, it is unclear how well they could individually explain item-wise free recall; this is the main goal of the present study. A key focus of this work is to conduct a broad comparison of psycholinguistic factors that may relate to word memorability, without a preconceived theory to support; for instance, Nairne et al. (2013) built upon Rubin and Friendly (1986) with an a priori emphasis on the influence of animacy on memory.

Conventional studies of verbal memory examine variability in a single word property in relation to memory recall while other properties are controlled for and held within a narrow range. Here, I use data from the Penn Electrophysiology of Encoding and Retrieval Study (PEERS) to examine word memorability by estimating free-recall probability for words from a database of 1,638 words, in a sample of 147 young adults. While a handful of studies have investigated the influence of individual word properties on free recall (e.g., Christian et al., 1978; Lau, Goh, & Yap, 2018; Rubin, 1980; Rubin & Friendly, 1986; Nairne et al., 2013), they did not consider the range of semantic properties examined here and were conducted in smaller databases of words. These findings were then replicated in a second data set (Lau et al., 2018) of 532 words from a sample of 116 young adults.

By examining the relative influences of different word properties in a large pool of words where the properties are more freely varied, we can gain a better understanding of how item properties influence memory.

## Method

### Data sets

#### Memory

Recall data were obtained from the Penn Electrophysiology of Encoding and Retrieval Study (PEERS; freely available at http://memory.psych.upenn.edu/Penn_Electrophysiology_of_Encoding_and_Retrieval_Study). PEERS is a large-scale memory study involving several experiments with slightly varying procedures. The study consisted of multiple experimental sessions of 12–16 lists each. In each list, 16 words were presented one at a time on a computer screen. Words were presented for 3,000 ms each, followed by an 800–1,200-ms intertrial interval. After the last word, there was a 1,200–1,400-ms delay between the offset of the last word’s presentation and the presentation of a tone and row of asterisks that indicated the beginning of the free recall test, where participants were given 75 s to vocally recall items from the list.

Lists had been constructed such that the same word was not presented more than once in a session and such that varying degrees of semantic relatedness occurred at both adjacent and distant serial positions. For some lists, participants were presented with a cue (font colour and typeface) that signalled an encoding task—either a *size* judgement (“Will this item fit into a shoebox?”), an *animacy* judgement (“Does this word refer to something living or not living?”), or no concurrent encoding task. Lists as a whole could either have a consistent encoding task (size, animacy, or none) or a mixture of size and animacy judgments. After the list presentation and free recall tasks, some sessions included a final free recall task, and all sessions then included a recognition test, neither of which were included in the analyses presented here, nor the EEG data that were also collected. Further details on the procedure are available in Lohnas and Kahana (2013), Healey and Kahana (2014), and Long, Danoff, and Kahana (2015). Here, I examined recall data from 147 young adult participants (ages 16–30 years) who each completed 20 sessions across the PEERS experiments.

The average (±*SD*) number of days between sessions was 4.11 (±1.59) days, ranging from 1 to 169 days; 33.3% of sessions were 2 days or fewer apart; 60.6% were 4 days or fewer apart; 94.7% were 10 days or fewer apart; 98.6% were 15 days or fewer apart. The average number of days between sessions was relatively consistent between sequential sessions (i.e., there was no clustering in how the sessions were distributed over time). The average (±*SD*) number of days between the first and last session was 78.04 (±30.18) days. 19.1% of participants completed all 20 sessions in 60 days or fewer; 95.9% completed in 110 days or fewer; 99.3% completed in 205 days or fewer—the single remaining participant completed the 20 sessions in 306 days.

**Fig. 1:**
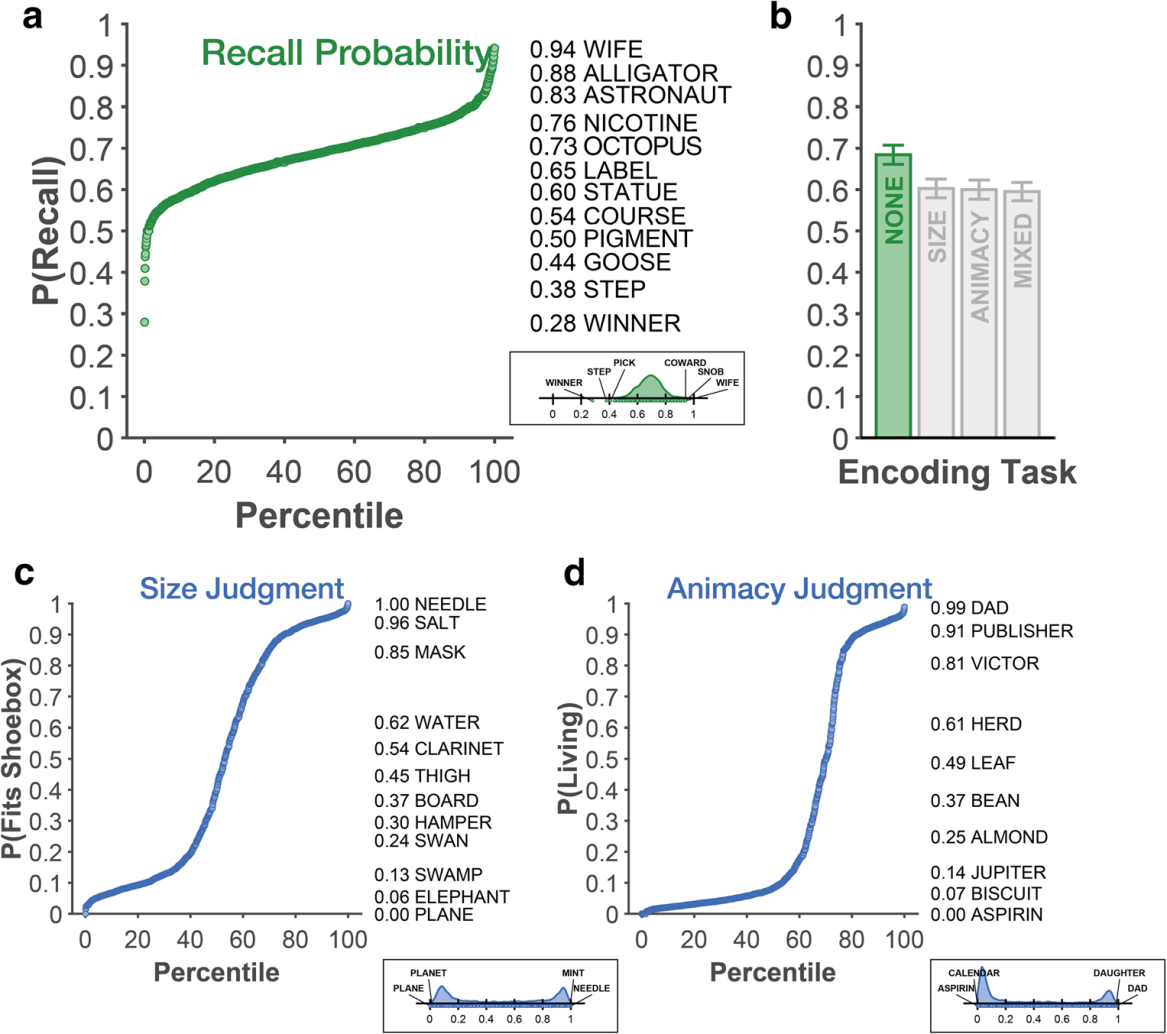
Response distributions for the (**a**) recall probability, as well as (**c**) size and (**d**) animacy judgments. Along with each distribution plot, words at different recall/judgement probabilities are listed to improve interpretation. Panel **b** shows the overall recall rates as a function of the list encoding task. Apart from a few noted exceptions, all recall analyses are based on the lists where no concurrent encoding task (here, “none”) was present, as such, this condition is highlighted in green. Error bars are 95% confidence intervals. (Colour figure online)

**Fig. 2:**
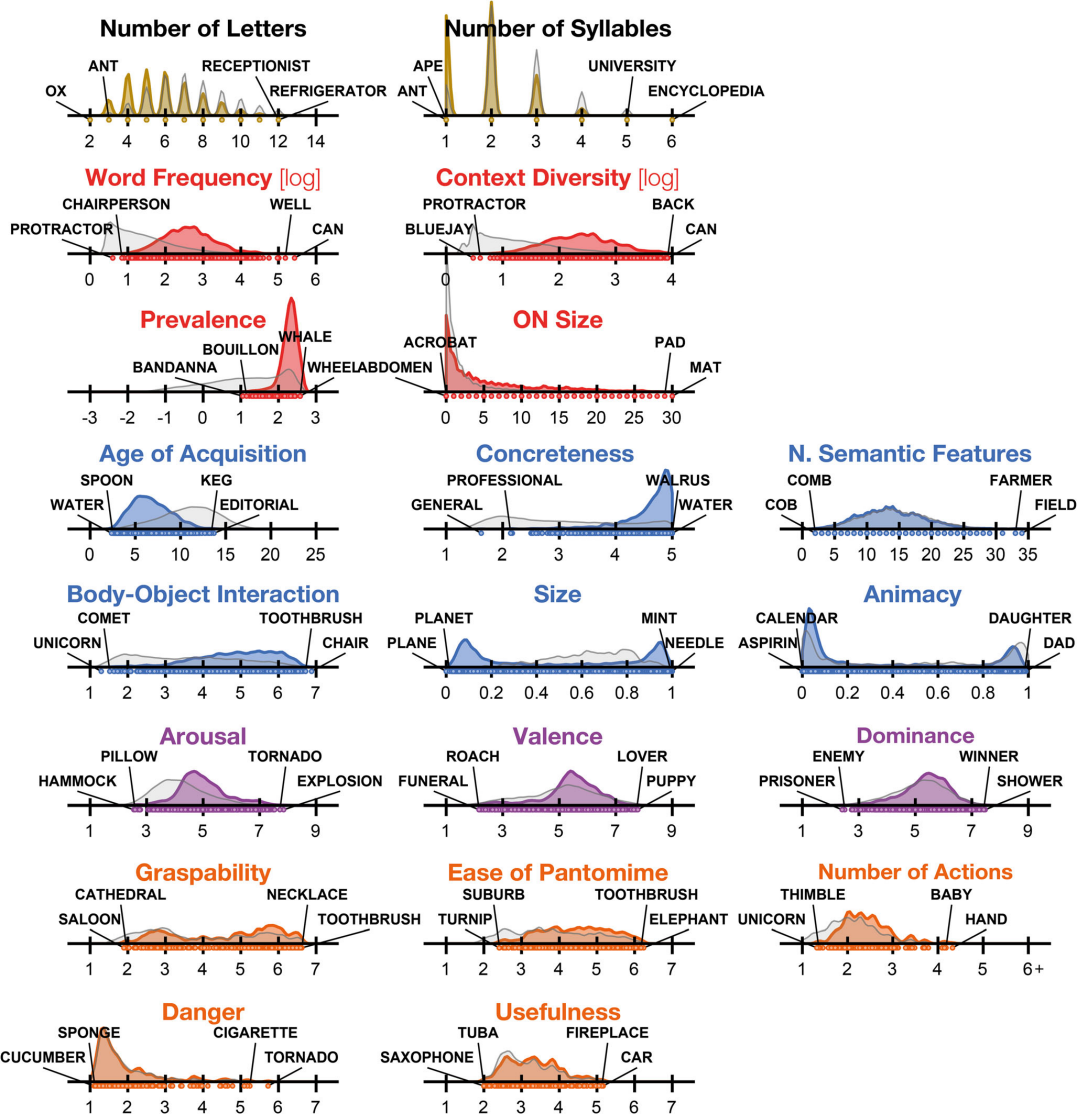
Rating density functions for all considered word properties using all available words. ON = orthographic neighbourhood. Ranges of each distribution determined based on the bounds of the rating scale or database min/max values. Dot plots below the *x*-axis show the specific values where words were present. Words on each end of the density distributions show the two highest and two lowest words for the respective word property. Colour of distributions is used to visually categorize the type of word property: yellow = length; red = lexical; blue = semantic; purple = affective; orange = function (subcategory of semantic, but also less words available). Reference distributions of the full available word databases (see main text) are overlaid in grey. See main text for detailed descriptions of each measure. (Colour figure online)

PEERS used 1,638 words. As described in Long et al. (2015), words were selected from the University of South Florida free association norms word database (Nelson, McEvoy, & Schreiber, 2004), based on their semantic relatedness and such that size and animacy judgments were plausible to be made for the words (i.e., are referents to physical objects; also see bimodal responses in Fig. 2). In the current study, ratings from the *size* and *animacy* judgments were also used as semantic word properties to be related to recall. The distribution of responses and example mean ratings for both judgments are shown in Fig. 1.

#### Word property databases

Many word properties were considered. While the MRC Psycholingustic Database (Wilson, 1988) includes many word properties, its values are relatively dated and less extensive than current databases. Along with distributions for subset of the 1,638 words where the word properties were available, Fig. 2 also shows the distribution for the word databases in their entirety (i.e., a reference distribution), to allow for a comparison between the words examined here and the possibility of their sampling imposing limits to how we consider the relationship between the given word property and the estimated word memorability. In most cases, Fig. 2 shows the entire range of possible values (e.g., ratings on a 7-point or 9-point Likert scale), but there are a few instances, noted when discussing the respective word property, where this was not the case.

*Number of syllables* were obtained with quanteda (Benoit et al., 2018), using the CMUdict database (Carnegie Mellon Speech Group, 2014), which has pronunciation information for more than 134 thousand words. Values were available for all 1,638 words. For the number of letter and syllable reference distributions, the entire CMUdict database was used, but was constrained to the range of values used in the 1,638 words. (Words in the database ranged from 1 to 33 letters and 1 to 14 syllables; in both cases, the longest word was SUPERCALIFRAGILISTICEXPEALIDOSHUS.)

*Word frequency* and *contextual diversity* counts were obtained from SUBTLEX (Brysbaert & New, 2009), which includes 60 thousand words and is based on a corpus of 16.1 million words extracted from subtitles from U.S. films and TV series. SUBTLEX was designed to supersede the Kučera and Francis (1967) norms, which have become dated and were based on a smaller corpus (1.014 million words). As is common for both measures, Log-10 transformed values will be used in the analyses. Counts were available for 1,606 of the 1,638 words. The range of word frequency and contextual diversity values had sufficiently similar ranges for the 1638 words in comparison to the full database. For word frequency, the min and max Log-10 values for the 1,638 words were [0.60, 5.43], while the database range was [0.48, 6.33]; for contextual diversity, the ranges were [0.48, 3.92] and [0.30, 3.92], respectively.

*Prevalence* ratings were obtained from Brysbaert, Mandera, McCormick, and Keuleers (2019), which includes 62 thousand words—largely the same as those in SUBTLEX (Brysbaert & New, 2009). Participants had to respond whether they knew the presented letter string or not, from lists of words and nonwords. For each word a percentage-known statistic was calculated and then probit transformed, such that the resulting scores follow a *Z* distribution. A prevalence score of 0 corresponds to 50% of participants knowing the word, whereas a score of +1.96 corresponds to 97.5% of participants. The range of percentages was truncated to 0.5% (−2.576) to 99.5% (+2.576). Counts were available for 1,624 of the 1,638 words.

*Orthographic neighbourhood size* was obtained using Westbury, Hollis, and Shaoul (2007), which has values for more than 111 thousand words. The measure is the number of different words that exist that are only one letter changed from the current word, while maintaining letter position (e.g., ONsize(MAT) = 30, corresponding to {BAT, CAT, EAT, …, MAN, MAP, MAW}). Values were available for all 1,638 words. The maximum orthographic neighbourhood size in the full database was 32 (MAG), with only three words exceeding 30.

*Age of acquisition (AoA)* ratings were obtained from Kuperman, Stadthagen-Gonzalez, and Brysbaert (2012), which includes 30 thousand words. Participants were asked to “enter the age (in years) at which [they] thought they had learned the word.” Participants could also respond that they did not know a word. Ratings were available for 1,613 of the 1,638 words. Since this included nearly all of 1,638 words, I did not use the more recent test-based AoA ratings of Brysbaert and Biemiller (2017) which are less continuous; though the measures are highly correlated (*r* = .76, as reported in Brysbaert & Biemiller, 2017).

*Concreteness* ratings were obtained from Brysbaert, Warriner, and Kuperman (2014), which includes nearly 40 thousand words. Brysbaert and colleagues provide a very clear definition of concreteness, beginning with, “Some words refer to things or actions in reality, which you can experience directly through one of the five senses. We call these words concrete words. Other words refer to meanings that cannot be experienced directly, but which we know because the meanings can be defined by other words. These are abstract words.” Ratings were made on a 5-point Likert scale, with 5 corresponding to concrete. Ratings were available for 1,617 of the 1,638 words.

*Number of semantic features* were obtained from Buchanan, Valentine, and Maxwell (2019), which includes more than 4 thousand words. This database includes the number of semantic features that are related to each word/concept (also see McRae, Cree, Seidenberg, & McNorgan, 2005). The beginning of the instructions was, “We want to know how people read words for meaning. Please fill in features of the word that you can think of. Examples of different types of features would be as follows: how it looks, sounds, smells, feels, or tastes; what it is made of; what it is used for; and where it comes from.” Of the 1,638 words examined here, some with the fewest semantic features (also referred to as semantic richness [see Tousignant & Pexman, 2012] or cue set size) were COB, COMB, TROUT; words with the most semantic features were FIELD, FARMER, COMPUTER. Ratings were available for 1,365 of the 1,638 words.

*Body–object interaction (BOI)* ratings were obtained from Pexman, Muraki, Sidhu, Siakaluk, and Yap (2019), which includes more than 9 thousand words. This database supersedes Tillotson, Siakaluk, and Pexman (2008), which included 1,618 words, though the databases are highly correlated (*r* = .87, as reported in Pexman et al., 2019). The beginning of the instructions was as follows: “Words differ in the extent to which they refer to objects or things that a human body can physically interact with. Some words refer to objects or things that a human body can easily physically interact with, whereas other words refer to objects or things that a human body cannot easily physically interact with.” Ratings were made on a 7-point Likert scale, with 7 corresponding to high BOI. Ratings were available for 1,461 of the 1,638 words.

*Affective* ratings (arousal, valence, and dominance) were initially obtained from Warriner, Kuperman, and Brysbaert (2013), which includes ratings for nearly 14 thousand words. This study collected ratings for three affective dimensions: emotional valence, arousal, and dominance. The beginning of the instructions was as follows: “The scale ranges from 1 (*happy* [excited; controlled]) to 9 (*unhappy* [calm; in control]). At one extreme of this scale, you are happy, pleased, satisfied, contented, hopeful [stimulated, excited, frenzied, jittery, wide-awake, or aroused; controlled, influenced, cared-for, awed, submissive, or guided]. When you feel completely happy [aroused; controlled] you should indicate this by choosing rating 1. The other end of the scale is when you feel completely unhappy, annoyed, unsatisfied, melancholic, despaired, or bored [relaxed, calm, sluggish, dull, sleepy, or unaroused; in control, influential, important, dominant, autonomous, or controlling]. You can indicate feeling completely unhappy [calm; in control] by selecting 9.” The valence and arousal scales were later reversed such that high values corresponded to happy and aroused, respectively. Ratings were available for 1,555 of the 1,638 words. In previously examining affective influences on recall, Long et al. (2015) collected arousal and valence ratings for all 1,638 words used in PEERS. These ratings were used instead, and were highly correlated with those from Warriner et al. (2013), arousal: *r*(1553) = .67, *p* < .001; valence: *r*(1553) = .92, *p* < .001. Nonetheless, the ratings from Warriner et al. were still used to estimate a reference distribution to compare the 1,638 words to.

Across all databases, the number of words where all 15 word properties was available were selected, resulting in a list of 1,185 words (from the full list of 1,638 words).

#### Function-related ratings

Heard, Madan, Protzner, and Pexman (2019) collected ratings for several semantic properties not present in other databases. This database was intended to examine how seven different motoric/function-related dimensions related to BOI and includes 621 words. These dimensions include: graspability (how easy can grasp object with one hand; also see Amsel, Urbach, & Kutas, 2012; Salmon, McMullen, & Filliter, 2010); ease of pantomime (how easily one can pantomime an object’s functional use so another can identify the object; also see Guérard, Lagacé, & Brodeur, 2015); number of actions (number of functional actions that can typically be performed with an object; also see Guérard et al., 2015); danger (how dangerous an object is for human survival; also see Wurm, 2007); and usefulness (how useful an object is for human survival; also see Wurm, 2007), as well as size and animacy. All measures were 7-point Likert scales, except for number of actions, which was instead a count from 1 to 6+. Higher values corresponded to more easy functional interaction, extremely dangerous, extremely useful, animate, and very large, respectively. However, ratings were available for only 253 of the 1,638 words. Since this is much less than the original word pool, the analyses using these ratings will be considered separately. Reassuringly, both the size, *r*(251) = .89, *p* < .001, and animacy, *r*(251) = .95, *p* < .001, were highly correlated between the PEERS ratings and those from Heard et al. Note that some discrepancy here is expected, as the PEERS participants rated both size and animacy as a yes/no response, whereas Heard et al. had participants make ratings on a 7-point Likert scale.

In addition to the five properties principally used from here (i.e., those plotted in orange in Fig. 2), the reference distribution for size was also estimated from this word database; animacy, however, was estimated from another database, detailed below.

#### Alternate animacy ratings

VanArsdall’s (2016) Study 1B collected normative ratings for 1,200 words across six animacy-related scales, available from Appendix C of the PhD dissertation. Though these data have not been published in an article, the norms here are available and serve as the most extensive set of animacy ratings available for comparison to those derived from the PEERS data set. Of these six scales, the living–nonliving scale was the most similar to the instructions used in PEERS; ratings were made on a 7-point Likert scale, with 7 corresponding to high living (VanArsdall, 2016, p. 161). Data for this measure were collected from 250 participants (after exclusions) recruited via Amazon MTurk, and each person rated a random selection of up to 120 words, presented as lists of 30 words; data for the other scales were obtained from other participants. The living ratings for the entire 1,200-word database were rescaled (i.e., PEERS ranged from 0 to 1, VanArsdall ranged from 1 to 7) and used as the reference distribution in Fig. 2.

A total of 957 words were included in both the PEERS and VanArsdall (2016) study, with the animacy/living ratings between the two studies highly correlated, Pearson’s *r*(955) = .97, *p* < .001; Spearman’s *ρ*(955) = .91, *p* < .001. It is also important to acknowledge that similar to the item ratings in PEERS, ratings in VanArsdall’s (2016) living scale were also quite bimodal; of the entire 1,200-word database, 496 words had mean ratings between 1 and 2 (high nonliving; 39.1%), while 402 words had mean ratings between 6 and 7 (high living; 33.5%), the remaining 329 words had ratings between 2 and 6 (27.4%). This bimodal distribution was by design, as VanArsdall (2016, p. 41) describes, an initial selection where words were chosen for the database such that approximately 36% each (430 words) should be “clearly living” and “clearly nonliving,” with the remaining 28% of items (340 words) to be more ambiguous.

## Results

### Item recall

Across all 147 participants, 42,762 lists of 16 words each were presented, yielding 684,192 words presentations. Across all 20 sessions, each participant completed lists involving no concurrent encoding task (44 or 52 lists, varied across PEERS experiments), size judgments (65 lists), animacy judgments (65 lists), or a mixture of both size and animacy judgments (112 lists); every session included all four types of lists. There was a total of 474,543 recall responses; of these responses, 419,351 were correct, yielding an average recall rate of 61.3%. As shown in Fig. 1b, recall differed based on the list encoding task, *F*(3, 438) = 150.0, *p* < .001, η_p_^2^ = .507, and was highest when no concurrent encoding task was used (all pair-wise Cohen’s *d*s > 1.0, *p*s < .001; also see Lohnas & Kahana, 2013), but did not differ across the remaining three encoding tasks.

Item recall, from the no concurrent encoding task lists, varied from as low as below 40% (WINNER, STEP, PICK) to as high as 94% (WIFE, SNOB, COWARD). Figure 1a shows the overall recall distribution from lists that had no concurrent encoding task, along with a sample of words and their respective recall probability and rank.

### Variance explained by individual word properties

Considering the variety of word properties considered here, results will be presented for two subsets of words: (1) all available words for the respective property; and (2) the 1,189-word subset where all main word properties were available. All results for individual word properties are shown in Table 1.

**Table 1:** Correlations (Spearman’s *ρ* [rho]) between word recall probability and individual word properties, using recall data from both PEERS and Lau et al. (2018); *p* values reported after Benjamini and Hochberg (1995) false discovery rate (FDR) correction for multiple comparisons. Correlations with corrected *p* values less than .05 are highlighted in **bold**. ON = orthographic neighbourhood

	PEERS				**1185 subset**		Lau et al. (2018)			
	**All Available Words**						**All Available Words**			
**Property**	*N. words*	*Mean (SD)*	*ρ [rho]*	*pFDR*	*ρ [rho]*	*pFDR*	*N. words*	*Mean (SD)*	*ρ [rho]*	*pFDR*
Number of letters	1,638	5.90 (1.85)	**.061**	**.022**	.038	.195	523	5.97 (1.96)	.000	.998
Number of syllables	1,638	1.84 (0.81)	**.077**	**.004**	.057	.052	523	1.84 (0.82)	.043	.378
Word frequency [log]	1,638	2.61 (0.67)	**.083**	**.002**	**.119**	**<.001**	512	2.41 (0.65)	**.201**	**<.001**
Context diversity [log]	1,638	2.37 (0.59)	**.059**	**.025**	**.092**	**.002**	512	2.17 (0.61)	**.185**	**<.001**
Prevalence	1,620	2.30 (0.21)	−.033	.210	−.032	.266	492	2.25 (0.26)	**.109**	**.031**
On size	1,638	4.93 (6.20)	**−.112**	**<.001**	**−.074**	**.011**	512	4.88 (6.35)	−.054	.282
Age of acquisition	1,613	6.73 (2.08)	**−.064**	**.019**	**−.092**	**.002**	489	6.34 (1.98)	**−.233**	**<.001**
Concreteness	1,617	4.60 (0.44)	.052	.052	.049	.092	489	4.82 (0.20)	**.133**	**.008**
N. semantic features	1,361	13.86 (4.99)	−.011	.718	−.021	.462	389	13.48 (5.02)	.096	.097
Body–object interaction	1,461	4.91 (1.02)	−.031	.263	**−.063**	**.030**	483	5.22 (0.89)	−.030	.567
Size	1,638	0.48 (0.37)	**−.239**	**<.001**	**−.250**	**<.001**	363	0.53 (0.36)	**−.196**	**.001**
Animacy	1,638	0.32 (0.37)	**.326**	**<.001**	**.353**	**<.001**	363	0.32 (0.38)	**.293**	**<.001**
Arousal	1,638	4.98 (0.83)	**.076**	**.005**	.055	.058	363	4.82 (0.82)	.077	.202
Valence	1,638	5.42 (0.96)	**.092**	**<.001**	**.111**	**<.001**	363	5.50 (0.82)	.114	.054
Dominance	1,555	5.37 (0.79)	**−.085**	**.002**	**−.081**	**.005**	466	5.43 (0.68)	−.072	.185
Graspability	253	4.60 (1.38)	−.085	.210			163	4.82 (1.29)	−.039	.653
Ease of pantomime	253	4.45 (0.92)	.003	.966			163	4.36 (0.97)	.104	.251
Number of actions	253	2.42 (0.53)	.109	.111			163	2.24 (0.47)	**.238**	**.006**
Danger	253	1.93 (0.89)	**.164**	**.018**			163	2.00 (1.03)	**.213**	**.014**
Usefulness	253	3.31 (0.69)	**.233**	**<.001**			163	3.29 (0.67)	**.335**	**<.001**

Since the words in PEERS were selected such that size and animacy judgments were both possible, some properties did not have much variability (see Table 1); for instance, all words were especially high in concreteness and prevalence, as well as moderately high in body–object interaction (BOI). Item distributions across all measures are shown in Fig. 2. Since the distribution for several word properties was substantially not-normal, Spearman’s *ρ* (rho) rank correlation was used.

**Fig. 3:**
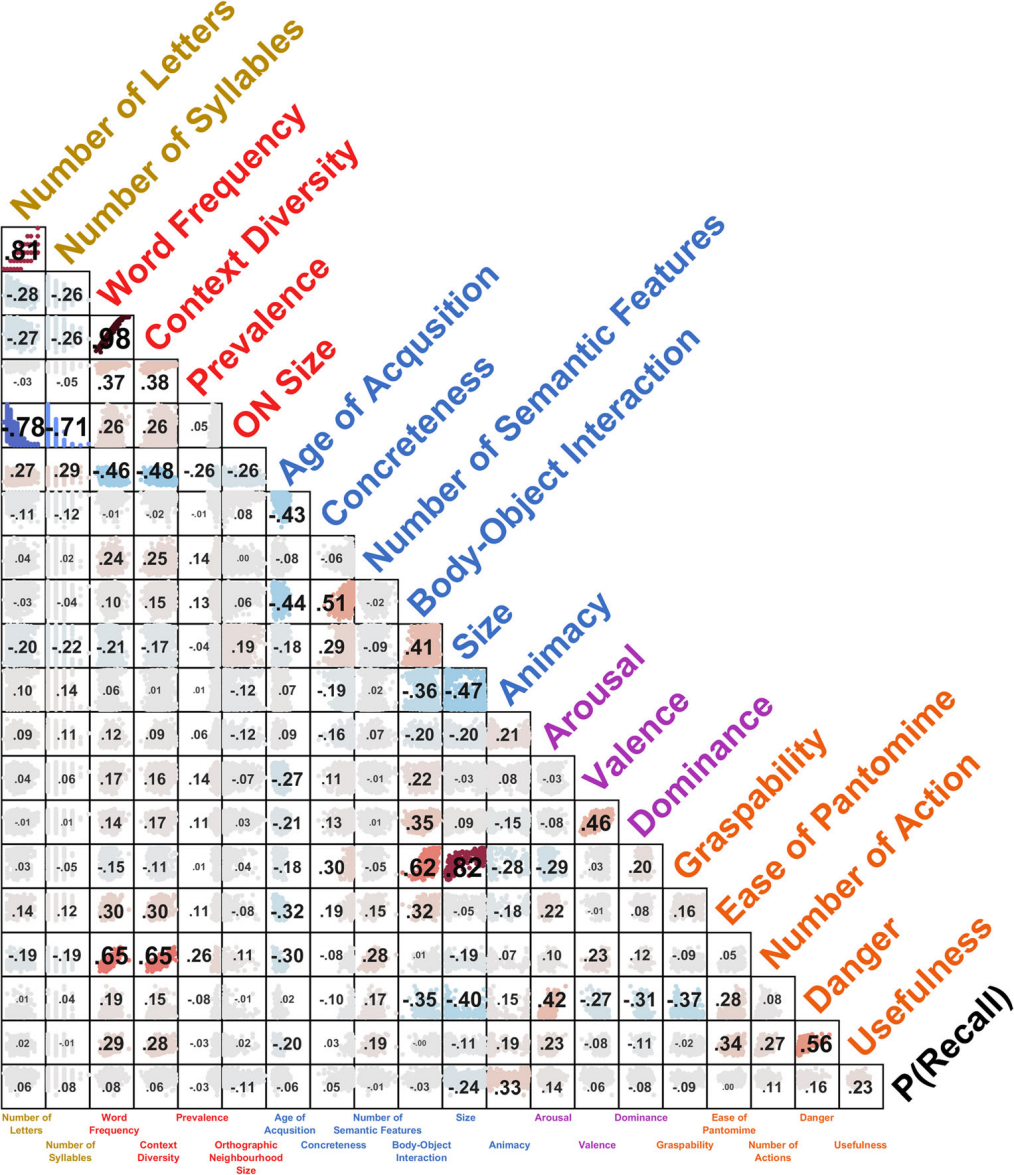
Spearman’s correlations between all word properties, using all available words from PEERS. Colour of word property labels is used to visually categorize the type of word property: yellow = length; red = lexical; blue = semantic; purple = affective; orange = function (subcategory of semantic, but also less words available). Correlation value text size and scatter plot colour correspond to the correlation value. See main text for detailed descriptions of each measure. (Colour figure online)

Correlations with recall probability indicate that animacy was by far the most relevant property for word recall—with better recall for animate word referents; words in the upper 10 percentile of animacy ratings had a 9.32% higher recall probability than those in the lowest 10 percentile. This was followed by size—with better recall for larger referents (5.99% difference in recall). Admittedly, animacy and size themselves are moderately correlated measures, *ρ*(1636) = −.465, *p* < .001 (also see Fig. 3). Nonetheless, as evaluated using partial correlations, both word properties explained a significant amount of unique variability in recall probability after controlling for the other property, animacy: *ρ*_*p*_(1,635) = .250, *p* < .001; size: *ρ*_*p*_(1,635) = −.104, *p* < .001. Since the item distributions for these two properties are bimodal (see Fig. 2), I wanted to rule out the possibility that the correlation was driven by merely a difference in recall rates for each mode (i.e., merely two levels of recall probability, one each for living vs. nonliving). As such, I conducted a median-split on the data, based on the word property of interest (i.e., animacy and size) and tested if the correlation was maintained in both halves of the data. Significant correlations were found for both halves of the animacy-recall analysis, below median: *ρ*(817) = .147, *p* < .001; above median: *ρ*(817) = .222, *p* < .001, but only the below median correlation was significant for the size analysis, below median: *ρ*(817) = −.255, *p* < .001; above median: *ρ*(817) = −.027, *p* = .44. Additionally, I extracted the middle two quartiles and tested if the relationship held for these intermediate, less extremely rated words. For both properties these correlations remained significant, though decreased in magnitude, animacy: *ρ*(816) = .134, *p* < .001; size: *ρ*(816) = −.088, *p* = .012.

Weaker, but nonetheless significant, correlations were then followed by arousal and word length (letters and syllables) measures, where higher arousal and longer words, respectively, were better recalled. See Fig. 3 for a correlation matrix of all word properties examined. Results were relatively consistent between the analyses based on all available words and the 1,185 subset, as shown in Table 1. Some lexical dimensions also performed well in explaining recall, particularly word frequency, orthographic neighbourhood size, and age of acquisition.

Several of the function-related properties from Heard et al. (2019) also performed quite well (which also included size and animacy). The magnitude of the correlations with danger and usefulness are particularly interesting, as one of possible explanation for the previous results with animacy and its robust effects across experimental designs (e.g., Bonin et al., 2015; Gelin et al., 2017; Nairne et al., 2013)—namely, that animacy is related to survival relevance and demonstrates the adaptive nature of memory. Considering that correlations of *r* > .20 have been shown to stabilize around *df* = 250 in mathematical simulations (Schönbrodt & Perugini, 2013), it would be ideal for future databases to prioritize expanding these ratings to a more extensive sample of words. If this is the case, danger and usefulness may be expected to perform even better than animacy. These findings indicate that further research using these semantic dimensions identified in the Heard et al. study would be prudent. A database aggregating all measures used here, for the 1,638 words, is provided in supplemental material.

### Further examination of semantic dimensions

An initial limitation of these results with respect to the size and animacy correlations is that the ratings were taken from the same sessions as when these judgments were collected at encoding. That is, even though the recall probabilities were calculated from the lists with no concurrent encoding judgment, participants may have been attending to these semantic dimensions to a greater degree since they were of particular relevance on other lists in the same session. For comparison, I also examined recall probabilities from the lists when those ratings were collected and observed an even stronger relationship between recall and animacy ratings, *ρ*(1636) = .62, *p* < .001, though the correlation with the size judgment was unchanged in magnitude, *ρ*(1636) = −.25, *p* < .001.

To obtain independent estimates of recall (i.e., that cannot be influenced by orienting task at encoding), I additionally examined the free recall data from Lau et al. (2018), which did not include this encoding judgment or examine these semantic dimensions in their item-analyses, based on 532 words in total. Here the words were selected to be concrete words from the McRae et al. (2005) semantic feature norm word database. Briefly, this study reported free recall rates collected from 116 participants (after exclusions); participants were presented with 28 lists of 19 words each, words were presented for 1.5 s. Here I found comparable results for the main findings (see right half of Table 1), animacy recall difference = 8.20%; size recall difference = 7.40%. Only 163 words from the Heard et al. (2019) study were included by Lau et al. (2018), but correlations were again notable for danger and usefulness. The animacy and usefulness correlations are higher in magnitude than any of word properties that had been considered in the analyses reported by Lau et al. (2018).

## General discussion

Here, I examined how various word properties relate to word memorability in free recall. These analyses exhaustively examined the influence of 20 lexical, semantic, and affective word properties on free recall performance. Importantly, in contrast to much of the prior literature on verbal memory, words varied along many dimensions, rather than specifically examining the influence of a single word property while others were constrained within a narrow range. The relationships between word properties and recall probability are demonstrated within a large database of 1,638 words (across 147 participants) and replicated in another database of 532 words (across 116 participants). In both cases, animacy was found to be highly relevant for better recall, along with two function-related properties (with respect to survival), danger and usefulness.

The finding that animacy was the word property most correlated with recall is consistent Nairne et al. (2013). While this result in comparison to *some* other word properties could have been predicted based on the findings of Nairne et al., the influence of other—not previously considered—embodied/functional perspectives of cognition were unclear. For instance, the relationship between body–object interaction (BOI) and memorability could have been motivated by sufficient theoretical arguments to make the case for an equal if not stronger influence of BOI on memory, as compared with animacy. However, results here indicate no meaningful influence of BOI on memory, at least with the words examined here—but the effects of animacy on memory are clear and converge with an existing literature.

The influence of animacy on cognition has been of interest for many decades, such as the foundational study by Heider and Simmel (1944) involving seemingly animate shapes engaging in social interactions. Much more recently, VanArsdall, Nairne, Pandeirada, and Blunt (2013) described nonwords with phrases associated with animate or inanimate properties (e.g., “loves to travel” vs. “filled with wires”) and found enhanced recognition and recall for the nonwords that had been associated with animate phrases. As is the case here, animacy can also be a preexisting semantic dimension, not just a property caused by the experimental presentation. Nairne et al. (2013) first drew explicit attention to animacy within the memory literature, drawing the connection that an adaptive memory system would prioritize processing of animate words due to the intrinsic association with survival. This animacy effect has been shown to generalize across a variety of experimental procedures (e.g., recall vs. recognition, words vs. pictures, different encoding instructions; Bonin et al., 2014; Bonin et al., 2015; Gelin et al., 2017). Later work has further built on this foundation to first suggest potential mechanisms (e.g., Bonin et al., 2015; Popp & Serra, 2016), though many of these have since been ruled out, such as being mediated by imagery (Gelin, Bugaiska, Méot, Vinter, & Bonin, 2019), emotional arousal (Meinhardt, Bell, Buchner, & Röer, 2018; Popp & Serra, 2018), or threat (Leding, 2019). Some studies have suggested that the memory enhancement due to animacy may relate to an attentional capture mechanism (e.g., Bugaiska et al., 2018; Gelin et al., 2017; Popp & Serra, 2016). Furthering our understanding of the basis of this animacy effect on memory is an ongoing topic of research.

An important consideration and potential limitation of the results presented here is that the words were not uniformly distributed across all dimensions. Figure 2 shows the overall word databases (or comparison databases) overlaid in grey to allow for a visual comparison of how the words examined in the current study compare to a broader potential pool. Most notably, concreteness and prevalence were higher than the reference distributions, as were word frequency and contextual diversity. Age of acquisition was also shifted towards earlier-acquired words. The remaining semantic, affective, and function properties were not as skewed relative to the respective reference databases. Animacy was similarly bimodal, even though the ratings were obtained from a wholly independent database; the size database was normed on a different scale and is less comparable. As a whole, these aspects of the word pool are important caveats to the presented findings—for instance, there were no words that were particularly low in frequency or concreteness, constraining their potential to explain memorability, particularly in comparison to studies that specifically studied these word properties. This consideration is needed to evaluate the generalisability of these results to other word sets and memory paradigms in the literature more broadly.

Though the various word properties were initially analyzed as their individual effects on memory, they include a complex pattern of inter-relations (as shown in Fig. 3). Reassuringly, these bivariate correlations replicate several prior findings. Number of letters and syllables are closely related (e.g., Baddeley et al., 1975; Hulme et al., 2004) and shorter words tend to have more orthographic neighbours (e.g., Glanc & Greene, 2012; Jalbert et al. 2011b). High arousal words have lower body–object interaction and semantic richness (Warriner et al., 2013). Moreover, some prior studies have indicated that specific word properties only influence recall when presented in pure lists, as opposed to the mixed lists used here, or only when another property is particularly high or low, but not at the other level. As the current goal was to compare a large set of properties and identify specific word properties that were relevant to recall probability, those more nuanced hypotheses were not evaluated here.

While several previous papers have examined free recall in relation to word properties from PEERS; for instance, word frequency in Lohnas and Kahana (2013) and emotion in Long et al. (2015) the relative influence of different word properties has not been compared. Though these studies focused on individual word properties and their influences on several memory measures (e.g., recall transition probabilities, task effects on recognition), none have considered a multitude of word properties to examine their relative influences on free recall. Further, it is important be considerate of where these data came from: young adults, who responded to recruitment flyers posted around the University of Pennsylvania campus, and who participated for a 20-session experiment. As such, it is likely that word memorability data will differ if obtained from another demographic, such as older adults or individuals from another locale. For a further discussion of sampling effects in behavioural research, see Henrich, Heine, and Norenzayan (2010).

In summary, here I found that semantic properties related to the referenced object and its functional use were the best performing dimensions in explaining word memorability, as measured by free-recall probability. Animacy performed the best of all considered word properties, in line with prior work highlighting the adaptive nature of memory (e.g., Nairne et al., 2013). This finding was then replicated using the recall data from Lau et al. (2018). The current results indicate that animacy is a highly relevant psycholinguistic dimension that is predictive of memory and should be a focus of further investigation. Other properties with functional features, such as danger and usefulness, are also ripe for further research.
